# Supplementary material for: Pediatric Lower Urinary Tract Dysfunction: A Comprehensive Exploration of Clinical Implications and Diagnostic Strategies
Source: Biomedicines. 2024 Apr 24;12(5):945. doi: 10.3390/biomedicines12050945 (PMC11118197; doi:10.3390/biomedicines12050945)
Supplement: Supplementary file 1 [file biomedicines-12-00945-s001.zip › biomedicines-2929549-supplementary.pdf]

Icahn School of Medicine at Mount Sinai

The Sol and Margaret Berger Department of Urology
Pediatric Urology

Dysfunctional Voiding Scoring System (DVSS)

Patient Name: ___________________________________ Date of Birth: _____________ Today's Date: ______________

Over the last month	Almost Never	Less than half the time	About half of the time	Almost every time	Not Available
1. I have had wet clothes or wet underwear during the night.	0	1	2	3	NA
2. When I wet myself, my underwear is soaked.	0	1	2	3	NA
3. I miss having a bowel movement every day.	0	1	2	3	NA
4. I have to push for my bowel movements to come out.	0	1	2	3	NA
5. I only go to the bathroom one or two times each day.	0	1	2	3	NA
6. I can hold onto my pee by crossing my legs, squatting or doing the "pee dance".	0	1	2	3	NA
7. When I have to pee, I cannot wait.	0	1	2	3	NA
8. I have to push to pee.	0	1	2	3	NA
9. When I pee it hurts.	0	1	2	3	NA
10. Parents to answer. Has your child experienced something stressful like the examples below?	No (0)			Yes (3)	
**Total**					

Examples:

New baby
New home
New school
School problems
Abuse (sexual/physical)
Home problems (divorce/death)
Special events (birthday)
Accident/injury
Other
